# Bowel perforation by a peritoneal dialysis catheter: report of two cases

**DOI:** 10.1186/s12882-017-0737-9

**Published:** 2017-10-16

**Authors:** Maki Fujiwara, Takeshi Soda, Takuya Okada, Hiroshi Kanamaru, Takahiro Inoue, Osamu Ogawa

**Affiliations:** 1 Department of Urology, Kitano Hospital, 2-4-20 Ohgimachi, Kita-ku, Osaka, 530-8480 Japan; 2 Department of Urology, Kyoto University Graduate School of Medicine, Kyoto, Japan

**Keywords:** Bowel perforation, PD catheter, SMAP, PWAT

## Abstract

**Background:**

Complications of peritoneal dialysis (PD) such as pain and catheter leakage are frequently reported. Delayed bowel perforation of a PD catheter is a rare adverse event but a serious complication associated with significant mortality. Bowel perforation of a PD catheter is difficult to differentiate from PD-related peritonitis and likely to result in a delay in diagnosis. Here, we report two cases of bowel perforation after PD catheter insertion by the stepwise initiation of PD using the Moncrief and Popovich technique (SMAP) and peritoneal wall anchor technique (PWAT).

**Case presentation:**

The first case was a 53-year-old woman with end-stage renal disease (ESRD) due to diabetic nephropathy and a history of entero-adhesiolysis. She underwent PD catheter insertion by the SMAP with PWAT. Four months after PD catheter insertion, the catheter was found to perforate sigmoid colon. The second case was a 57-year-old woman with ESRD due to large polycystic kidney disease. She underwent the same procedure. After exteriorization of the catheter, she developed peritonitis due to perforation of the catheter tip into the bowel. Both patients were safely removed the catheter with uneventful recovery.

**Conclusion:**

We reported two cases of a rare complication of PD catheter. The SMAP method, PWAT, enlarged kidneys and migration of the lower cuff may be risk factors of bowel perforation of a PD catheter.

## Background

Peritoneal dialysis (PD) has been widely used for its safety and efficacy. Complications such as pain and catheter leakage are the problems most frequently reported. Erosion of the PD catheter into the bowel is a rare complication, and patients experience serious consequences such as peritonitis, difficulty in draining, and feculent dialysate effluent with watery diarrhea [1]. Kagan, et al. reviewed the publications from 1980 to 1995 and delayed bowel perforation is associated with a 29% mortality [2]. Perforative peritonitis in a patient undergoing PD is often difficult to diagnose because free air and ascites are not specific. Especially, some cases of free air are known to introduce air with dialysate infusion due to recurrent technique error. Here, we report two cases of bowel perforation after PD catheter insertion using the stepwise initiation of PD using the Moncrieff and Popovich technique (SMAP) with peritoneal wall anchor technique (PWAT). We compared our patients with the previous cases and discussed underlying risk factors of the rare complication.

## Case presentation

### Case 1

**Fig. 1:**
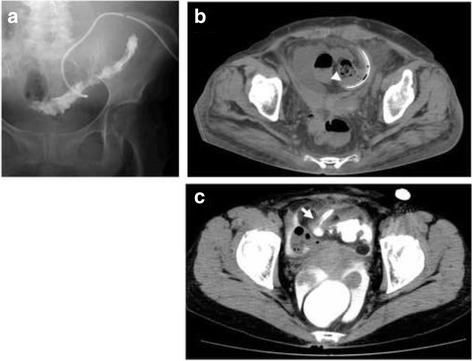
**a** Contrast catheterography showed accumulation of the contrast agents in the sigmoid colon. **b** The bowel perforation of PD catheter (arrowhead) was seen on computed tomography scan. **c** Computed tomography scan with contrast agents into the catheter (arrow) showed accumulation of the contrast agents in the colon and rectum

A 53-year-old woman with end-stage renal disease (ESRD) due to diabetic nephropathy and a history of entero-adhesiolysis due to endometriosis underwent laparoscopic-assisted double-cuffed swan neck catheter insertion. This was achieved by the SMAP with PWAT. Severe adhesion of the omentum and bowels was present at the operation and we performed adhesiolysis. Postoperative recovery was uneventful. Four months later, exteriorization of the external segment of the catheter was performed to start PD. The drainage had a foul smell. Before starting PD, drainage was poor with cloudy brown fluid. Contrast catheterography showed accumulation of contrast agents in the sigmoid colon without overflow into the peritoneal cavity (Fig. 1a, b). Open surgery revealed a mature tract between the insertion site of the catheter and the sigmoid colon. The catheter was covered by peritoneal mesothelium and the fistula site was covered by omentum. The catheter was removed and the tract was closed with sutures. The patient recovered uneventfully with hemodialysis.

### Case 2

A 57-year-old woman with ESRD due to large polycystic kidney disease underwent laparoscopic-assisted double-cuffed swan neck catheter insertion by the same method described above and had no signs of peritonitis for seven months. After the exteriorization, fever and lower abdominal pain developed. Instillation of dialysis solution via catheter led to severe watery diarrhea. A CT scan after contrast catheterography showed accumulation of the contrast agents in the colon and rectum (Fig. 1c). Open surgery revealed fistula formation between the sigmoid colon and the side of the surface of the catheter. The catheter was removed and colostomy was performed. She made a rapid postoperative recovery with permanent hemodialysis.

## Conclusion

Delayed bowel perforation of a PD catheter is an uncommon complication. Patients often present with peritonitis and watery diarrhea at the time of PD inflow. However, patients do not necessarily present with symptoms, especially if bowel erosion occurs by dormant PD catheters. Thus, prompt decision to perform exploratory laparotomy in patients suspected of having endogenous peritonitis is critical.

From 2006 to 2015 we performed 123 PD catheter insertions. Thirty-two cases received SMAP, 53 cases received PWAT and 24 cases received both methods. Among 24 patients implanted using both methods, 2 cases had bowel perforation. But we did not experience the complication among the remaining 99 cases. Brown, et al. who used SMAP, but not using PWAT, found that bowel perforation was extremely rare with only two cases (0.5%) in 435 patients. Thus, his findings suggest that SMAP method in itself might not induce bowel perforation [3]. Additionally, Rubin et al. reported that bowel perforation occurred in 0.1% with conventional catheter implantation techniques, that is, neither SMAP nor PWAT was used [4]. Kagan, et al. and Chu, et al. speculated that dormant catheters are more likely to erode the bowel [2, 5]. A long duration of a PD catheter in the abdominal cavity without peritoneal fluid, which bathes the bowel loops acting as a barrier of adhesion of the catheter to the bowel wall, increases the risk of pressure-induced necrosis by the immobile catheter. Review of the other cases in the literature indicated that half were attributable to an unused PD catheter, typically 1.6–48 months after use had ceased [6]. PWAT is a simple method of fixing the catheter on the abdominal wall to prevent catheter malposition [7]. This method might create a predisposition to impingement of bowel loops by catheter that subsequently led to pressure erosion and perforation. After we stopped using SMAP and PWAT during the PD catheters insertion, delayed bowel perforation hasn’t occurred thereafter. Therefore, we suggest that a delayed perforation is the result of continuous pressure necrosis from the catheter tip on the bowel wall initiated by the unused peritoneal catheter due to the SMAP and fixing the catheter tip due to the PWAT.

In addition, several potential risk factors for perforation were noted. Enlarged kidneys due to polycystic kidney disease occupying most of the abdominal cavity space might result in elevated intra-abdominal pressure [8]. This might induce indolent erosion of PD catheters into the bowels in Case 2. When the lower cuff of the double-cuffed catheters migrates into the peritoneal cavity, adhesion of the cuff to the intestinal wall may be another mechanism of bowel perforation [9]. However, this didn’t play any role in our patients.

In conclusion, physicians should be aware that the SMAP, PWAT, and elevated intra-abdominal pressure caused by such as enlarged kidneys may be risk factors of bowel perforation of a PD catheter.

## Abbreviations

CT: computed tomography
ESRD: end-stage renal disease
PD: peritoneal dialysis
PWAT: peritoneal wall anchor technique
SMAP: stepwise initiation of PD using the Moncrief and Popovich technique
